# Risk of Thyroid Nodular Disease and Thyroid Cancer in Patients with Acromegaly – Meta-Analysis and Systematic Review

**DOI:** 10.1371/journal.pone.0088787

**Published:** 2014-02-14

**Authors:** Kosma Wolinski, Agata Czarnywojtek, Marek Ruchala

**Affiliations:** Department of Endocrinology, Metabolism and Internal Medicine, Poznan University of Medical Sciences, Poznan, Poland; Endocrine Research Center (Firouzgar), Institute of Endocrinology and Metabolism, Iran (Islamic Republic Of)

## Abstract

**Introduction:**

Acromegaly is a quite rare chronic disease caused by the increased secretion of growth hormone (GH) and subsequently insulin - like growth factor 1. Although cardiovascular diseases remains the most common cause of mortality among acromegalic patients, increased prevalence of malignant and benign neoplasms remains a matter of debate. The aim of this study is to evaluate the risk of thyroid nodular disease (TND) and thyroid cancer in patients with acromegaly.

**Materials and Methods:**

PubMed, Cochrane Library, Scopus, Cinahl, Academic Search Complete, Web of Knowledge, PubMed Central, PubMed Central Canada and Clinical Key databases were searched to identify studies containing. Random–effects model was used to calculate pooled odds ratios and risk ratios of TND in acromegaly. Studies which not included control groups were systematically reviewed.

**Results:**

TND was more frequent in acromegaly than in control groups (OR = 6.9, RR = 2.1). The pooled prevalence of TND was 59.2%. Also thyroid cancer (TC) proved to be more common in acromegalic patients (OR = 7.5, RR = 7.2), prevalence was 4.3%. The pooled rate of malignancy (calculated per patient) was equal to 8.7%.

**Conclusions:**

This study confirms that both TND and TC occur significantly more often in acromegalic patients than in general population. These results indicate that periodic thyroid ultrasound examination and careful evaluation of eventual lesions should be an important part of follow-up of patients with acromegaly.

## Introduction

Acromegaly is a rare chronic disease caused by the increased secretion of growth hormone (GH) and subsequently insulin-like growth factor 1 (IGF-1) [1], [2]. Cardiovascular diseases are very common and remain the most common cause of mortality among acromegalic patients [2], [3]. However, increased prevalence of malignant and benign neoplasms is also a matter of debate [2], [4]. Most studies were focused on colorectal and thyroid tumors, however also elevated risk of other, e.g. breast, central nervous system, adrenals or urinary tract neoplasm were reported [2], [4], [5], [6], [7], [8]. Meta-analysis performed by Rokkas et al. [5] proved the increased risk of colon cancer. The issue of benign and malignant thyroid tumors is not as well established as there was no meta-analysis on the topic and outcomes of particular studies were dispersed.

The aim of this study is to evaluate the risk of thyroid nodular disease (TND) and thyroid cancer (TC) in patients with acromegaly and also to combine results of the studies including control groups to assess if the risk is significantly higher than in general population.

## Materials and Methods

### Selection of the Studies

We have searched the PubMed/MEDLINE, Cochrane Library, Scopus, Cinahl, Academic Search Complete, Web of Knowledge, PubMed Central, PubMed Central Canada and Clinical Key databases from January 1960 up to May 2013 in order to find all relevant journal articles. We have used the search term: acromegaly and (thyroid or “thyroid cancer” or “thyroid nodules” or goitre). Only full-text journal articles written in English were taken into account. We have also searched manually the references of review articles in order to avail eventually omitted studies. Two researchers (K.W., A.C.) searched all included databases independently and prepared list of included studies. In case of discrepancies between lists, authors were reading doubtful articles together.

### Data Extraction

We have recorded data on study design, year of publication, country of origin, number of the patients, sex and age of participants, duration of the disease, methods of the thyroid examination (e.g. ultrasonography, palpation), number of patients with and without thyroid lesions and with thyroid cancer. In case of studies including control group, same data on this group were recorded. Studies with control group matched by age and sex was included. Studies with control groups not matched by this parameters were excluded to avoid within study bias.

### Statistical Analysis

We have meta-analyzed odds ratios (OR) and risk ratios (RR) using a random – effect model using Statistica v.10 software with medical package. Heterogeneity between studies was assessed using the Q statistics and I2 statistics. Q and i^2^ values given in “Results” are based on the odds ratio calculations. If calculation of OR was impossible due to zero cells, a constant (0.5) were added to all columns. Publication bias was assessed using Kendall’s tau. If publication bias was present we performed cumulative metaanalysis and also re-performed calculations with exclusion of the studies with highest standard error. We used also data from all included studies (with and without control groups) to calculate the pooled prevalence of thyroid nodular disease (TND) and thyroid cancer (TC) as well as malignancy rates. These data were meta-analyzed using random – effect model according to the methodology described by Borenstein et al. [9]. Only studies assessing the thyroid by ultrasonographic (US) examination were included in calculations concerning TND. Other articles (e.g. about palpable nodules only) have been systematically reviewed.

## Results

### Case – control Studies

**Figure 1:**
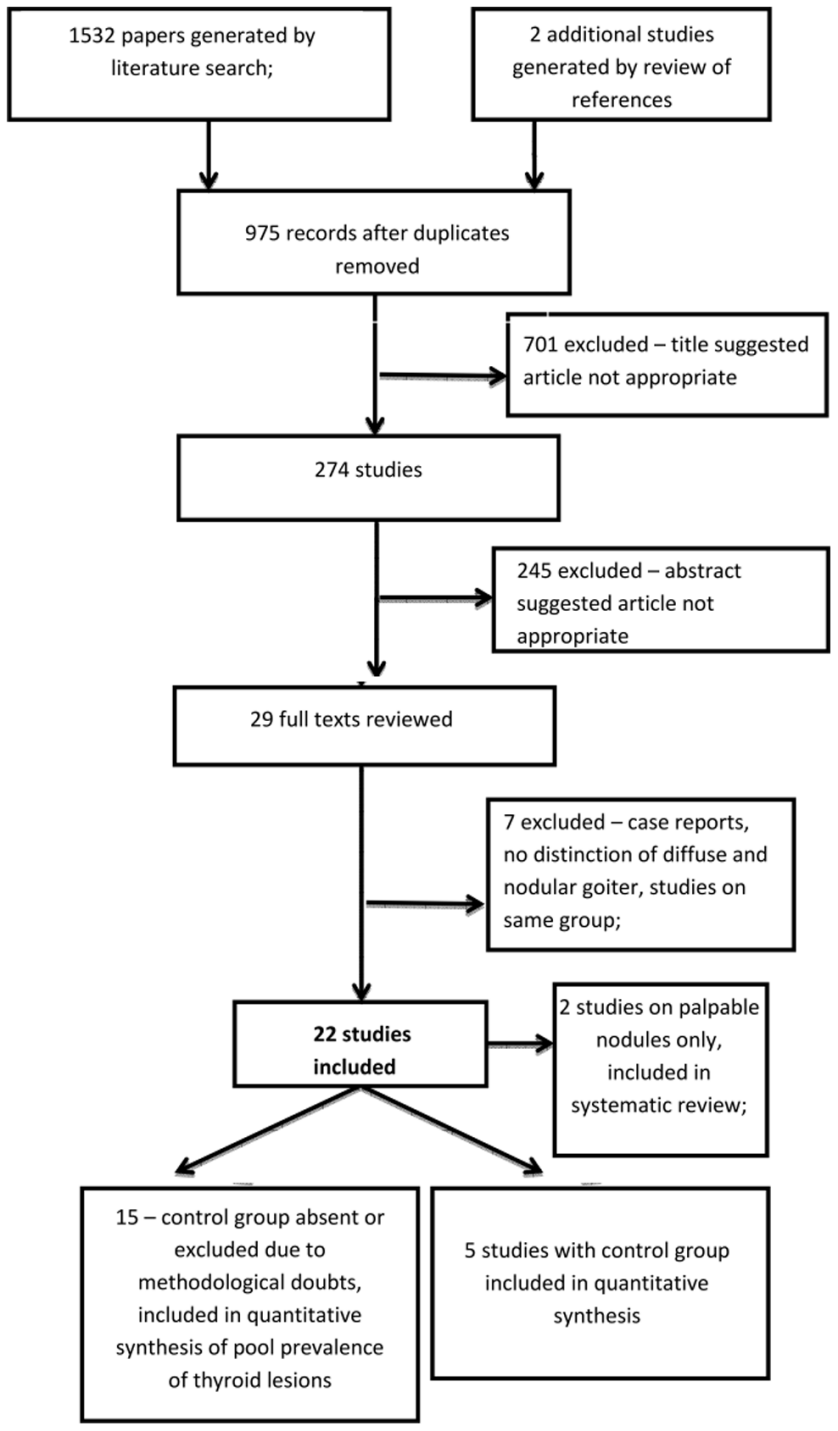
Flowchart presenting the steps of literature search and selection.

The search results and steps of selection are shown on the flowchart (figure 1). Nine studies including control group were identified. In one of them [10] only palpational examination of thyroid was performed. In one study [11] the control group was not matched by age and BMI, in another one – such details about control group were not given [12]. In study performed by Cannavo et al. [13] control group was not matched by sex. These four studies were excluded from the meta-analysis. Another five studies, two prospective and three retrospective have been included. [table 1] In case of 15 studies only data on prevalence of thyroid lesions or thyroid cancer were available (without data on the control groups). These studies were included in quantitative synthesis of pool prevalence of thyroid lesions. Two studies contained data on palpable nodules only; these studies were systematically reviewed.

**Table 1:** General characteristic of case – control studies on the frequency of thyroid nodules and thyroid cancer in patients with acromegaly.

Study	Year	Country	Patients	Mean age;	Control group	Mean follow-up	Comments
dos Santos et al. [14]	2012	Brasil	76 women, 48 men	45.1, SD = 13.4	263, not specified		
Hermann et al. [15]	2004	Germany	39 women, 34 men	55, SD = 13	199, healthy volunteers	7.3, SD = 4.1	Retrospective
Gasperi et al. [16]	2002	Italy	147 women, 111 men	50, SD = 13	150, non-functioning or PRLsecreting adenomas		
Popovic et al. [17]	1998	Yugoslavia	137 women, 83 men	49.5, SD = 0.9^1^	248, non-functioning or PRLsecreting adenomas	4.5, SD = 0.4	Retrospective
Barzilay et al. [18]	1991	USA	43 women, 44 men	Median 37^1^	198, non-functioning or PRLsecreting adenomas	Median - 13	Retrospective; data on TND not included – no distinction between nodular and diffused goiter;
Cannavo et al. [13]	2000	Italy	17 women, 11 men				Control group not matched by sex;
Cheung et al. [11]	1997	Australia	16 women, 21 men	49.5, SD = 14.5	37, hospital workers	9.9^1^	Control group not matched by BMI and age; not included into meta-analysis;
Junik et al. [12]	1997	Poland	18 women, 21 men	42, SD = 8	98 healthy volunteers		Mean age of control group not given;
Wüster et al. [10]	1991	Germany					Patients examined by palpation only; not included into meta-analysis;

1 estimated duration of acromegaly.

Abbreviations: SD – standard deviation; TND – thyroid nodular disease; BMI – body mass index.

**Figure 2:**
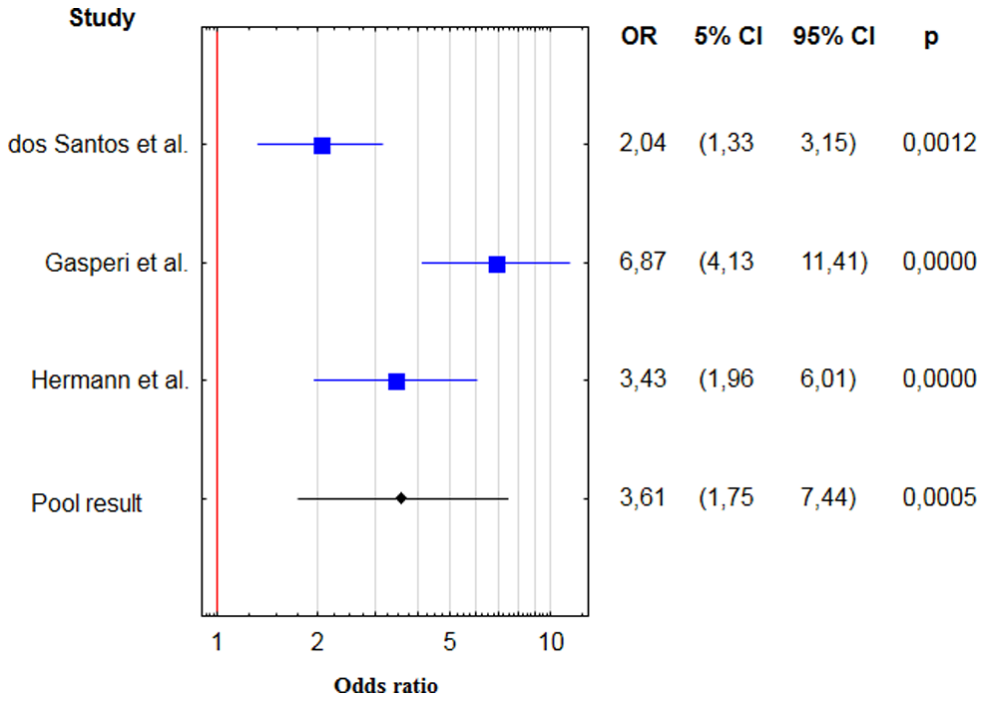
Forest plot showing individual and pooled ORs with 95% CI and p - values for studies comparing the prevalence of thyroid nodular disease in acromegalic patients and control groups.

For thyroid nodules the pooled OR was 3.6 with 95% confidence interval (CI) 1.8–7.4 [fig. 2, table 2], RR = 2.1, 95% CI 1.3–3.3. There were no evidences for significant heterogeneity (Q = 1.8, degrees of freedom (df) = 2, p value = 0.40; i2 = 0.0%). There is no evidence for publication bias (Kendall’s tau = 0.33, two – tailed p value = 0.60).

**Table 2:** Results of case – control studies containing data on frequency of thyroid nodular disease in patients with acromegaly.

Study	Patients with TND	Patients without TND	Control group – TND	Control groupwithout TND	OR
dos Santos et al. [14]	67	57	96	167	2.0 (1.3–3.2)
Hermann et al. [15]	46	27	66	133	3.4 (2.0–6.0)
Gasperi et al. [16]	143 (including 37 toxic nodular goiter)	115	23	127	6.9 (4.1–11.4)
**Total (random effect model)**					**3.6 (1.8–7.4)**

Abbreviations: SD – standard deviation; TND – thyroid nodular disease; OR – odds ratio.

**Figure 3:**
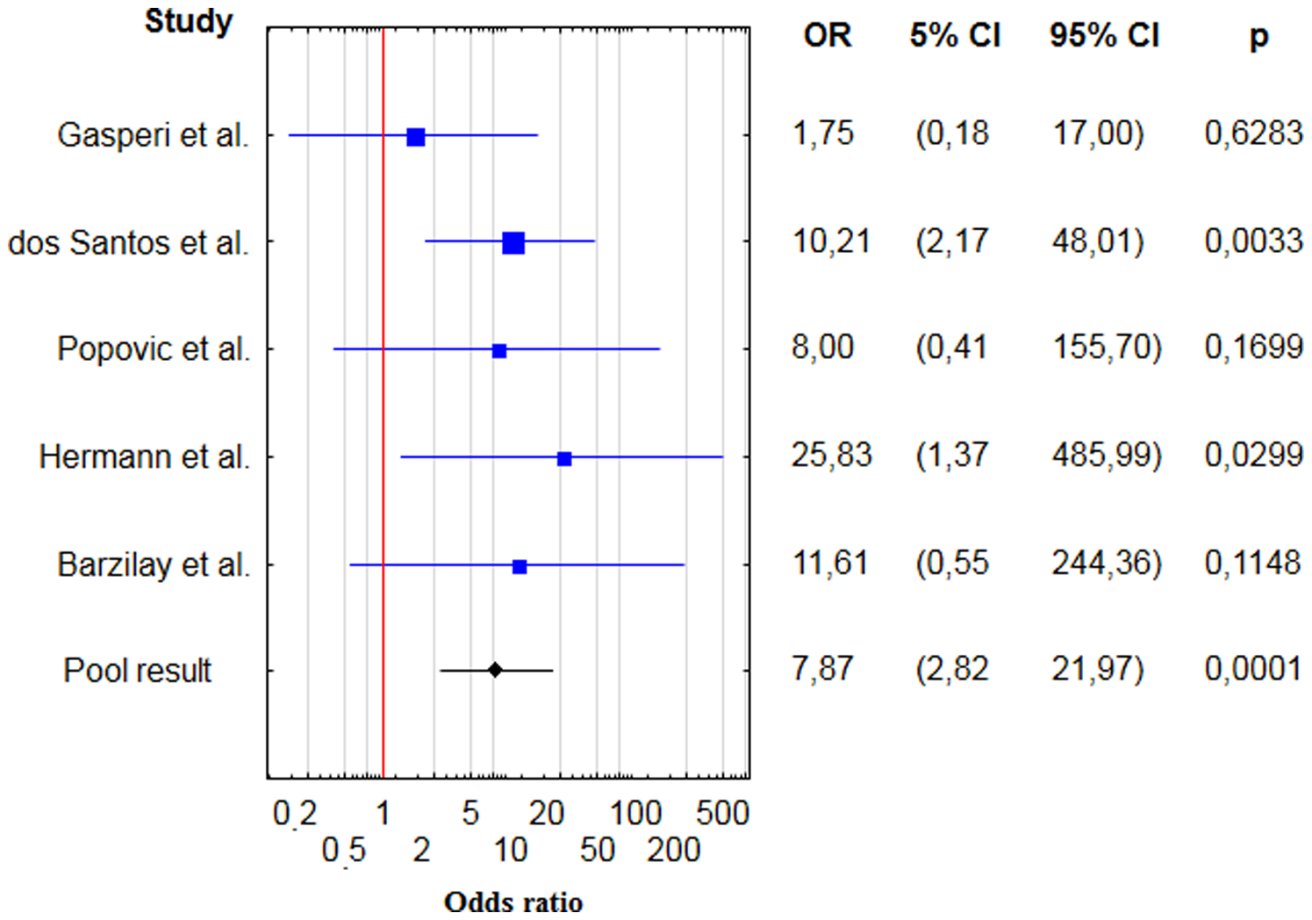
Forest plot showing individual and pooled ORs with 95% CI and p - values for studies comparing the prevalence of thyroid cancer in acromegalic patients and control groups.

**Figure 4:**
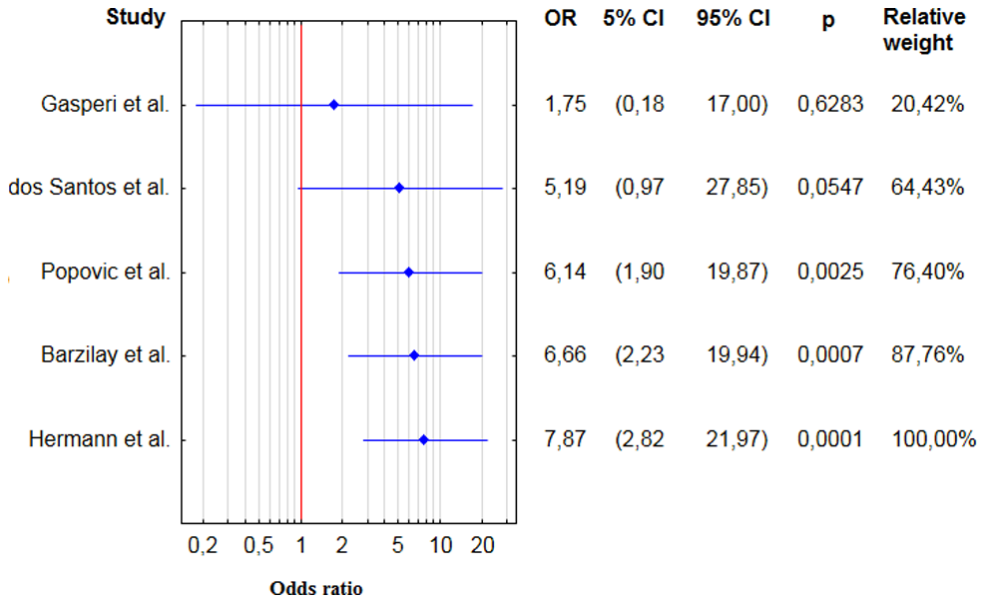
Cumulative forest plot for studies comparing the prevalence of thyroid cancer in acromegalic patients and control groups.

For thyroid cancers the pooled OR was 7.9 (95% CI 2.8–22.0) [fig. 3, table 3], RR = 7.6 (95% CI 2.7–20.8). There are no evidences for significant heterogeneity (Q = 2.5, degrees of freedom (df) = 4, p value = 0.65; i2 = 0.0%). There is no evidence for publication bias (Kendall’s tau = 0.80, two – tailed p value = 0.05). However, the calculations of publication bias was of borderline statistical significance. Exclusion of the study with the highest standard error [15] would slightly decrease the pooled result (OR 6.7) and it would eliminate this borderline publication bias (Kendall’s Tau 0.67, p = 0.17). Cumulative metaanalysis was shown on figure 4 [figure 4].

**Table 3:** Results of case – control studies containing data on frequency of thyroid cancer in patients with acromegaly.

Study	Patients with TC	Patients without TC	Control group – TC	Control group– without TC	OR
dos Santos et al. [14]	9	115	2	261	9.5 (2.2–48.0)
Hermann et al. [15]	4	69	0	199	25.8 (1.4–486.0)
Gasperi et al. [16]	3	255	1	149	1.7 (0.2–16.9)
Popovic et al. [17]	3	217	0	248	8.0 (0.4–155.7)
Barzilay et al. [18]	2	85	0	198	11.6 (0.6–244.4)
**Total**					**7.9 (2.8–22.0)**

Abbreviations: SD – standard deviation; TC – thyroid cancer; OR – odds ratio.

### Studies without Control Group

Results of studies which did not include control group or included groups which were excluded from the meta-analysis according to some methodological doubts (e.g. control group not matched by age) are shown in table 4 [table 4].

**Table 4:** Studies without control group or with control group exluded from meta-analysis.

Author	Year	Country	Patients	Age	Patients with TND	Patients with TC	% of malignantnodules	Duration of the follow-up^1^
**Prospective**								
Rogozinski et al. [19]	2012	Argentina	22 women, 12 men	Median –55	23 (67.6%)	4 (11.8%)	17.4%	
Gullu et al. [20]	2010	Turkey	60 women, 45 men(thyroid US performedin 100 patients)	47.9, SD = 11.5	62 (62.0%)	5 (5.0%)	8.1%	13.02, SD = 7.1
Cheung et al. [11]	1997	Australia	16 women, 21 men	49.5, SD = 14.5	16 (43.2%)			9.9^3^
Junik et al. [12]	1997	Poland	18 women, 21 men	42, SD = 8	18 (46.2%)			
**Retrospective**								
Anagnostis et al. [21]	2011	Greece	70 women, 45 men	47, SD = 14	85 (74.1%)			8.8, SD = 0.8
Baldys-Waligórska et al. [22]	2010	Poland	71 women, 30 men	51.8, SD = 15.4	64 (63.0%)	3 (2.9%)	4.7%	9.4, SD = 6.5
Ruchala et al. [23]	2009	Poland	52 women, 34 men	49.9, SD = 11.1	65 (75.6%)	5 (5.8%)	7.7%	
Kurimoto et al. [24]	2008	Japan	86 women, 54 men,thyroid US in 83 patients	55, SD = 25	62 (74.7%)	4 (4.8%)	6.5%	
Bolanowski et al. [25]	2006	Poland	75 women, 55 men	women - 52.6,men –51.6		1 (0.8%)		Women –10.5, men –12.0
Tita et al. [26]	2005	Italy	70 women, 55 men	49.9^2^	72 (57.6%)	9 (7.2%)	12.5%	Median 8.2
Cannavo et al. [13]	2000	Italy	17 women, 11 men	51.1, SD = 11.2	14 (50.0%)			14.2, SD = 7.5^3^
Higuchi et al. [27]	2000	Japan	19 women, 25 men	women: 50.9,men: 53.3		2		women: 7.5 men: 5.3,
Kasagi et al. [28]	1999	Japan	26 women, 22 men^5^	46.7, SD = 12.2	16 (43.2%)	2 (5.4%)	12.5%	
Nabarro et al. [29]	1987	UK	123 women,133 men		27^4^			6.8
**Register - based**								
Mestron et al. [30]	2004	Spain	741 women, 478 men	45^2^		2		
Baris et al. [31]	2001	Sweden, Denmark	888 women, 746 men	60.7		3 (SIR^ = ^4.3)		Sweden –10.3, Denmark –9.0
Orme et al. [32]	1998	UK	1239			1 (SIR = 2.5)		
Ron et al. [33]	1991	USA	1041 men			1 (SIR = 4.3)		

1 data were included only when it was clearly reported if given time was the time since diagnosis or since estimated onset of the disease;

2 at the time of diagnosis;

3 estimated time of duration of the disease;

4 Palpable nodules only.

5 11 patients examined only by palpation were excluded; descriptive statistics refer to the whole group;

Abbreviations: SD – standard deviation; SIR – standarized incidence ratio; TC – thyroid cancer; TND – thyroid nodular disease.

Eleven studies were included. Using also the data about prevalence from case – control studies, there were 13 papers containing data on TND frequency in ultrasound (US) examination and also 13 bringing data on thyroid cancer occurrence. Two further studies contained information about palpable thyroid nodules.

Prevalence of thyroid lesions fluctuated from 43.2% to 75.6% in US examination. In total there were 668 patients with and 457 without TND included. The pooled prevalence meta-analyzed using random – effect model is equal to 59.2% with 95% CI 52.7% –66.5%.

In two studies about the prevalence of palpable thyroid nodules was given in two papers and it was 38.8 and 10.5%.

Prevalence of TC fluctuated from 0.8% to 11.8%. In total there were 55 patients with and 1317 without TC included. The pooled prevalence meta-analyzed using random – effect model is equal to 4.3% with 95% CI 3.0% –6.2%.

### Register – based Studies

Four studies based on registers of acromegalic patients and cancer patients were identified [table 4].

### Malignancy Rate in Thyroid Nodules

Ten studies included data both on thyroid nodules and thyroid cancer frequency what allows to calculate the risk of malignancy in acromegalic patients with TND. There were 620 patients with TND including 48 malignancies. The pooled rate of malignancy (calculated per patient) meta-analyzed using random – effect model is equal to 8.7% with 95% CI 6.1% –12.3%. Comparing the risk of malignancy in the studies containing control group, the RR of malignancy in patients with TND and acromegaly was insignificantly higher than in patients with TND and without acromegaly – RR = 3.2, 95% CI 0.5–20.1.

## Discussion

Thyroid nodular disease turned out to be significantly more frequent in patients with acromegaly than in control groups (OR = 3.6, RR = 2.1) and it seems to be a very common disorder in these patients (prevalence slightly below 60%). According to Wüster et al. [10] also palpable thyroid nodules occurs significantly more often in acromegalic patients TC also proved to be more common in acromegaly (OR = 7.9, RR = 7.6), however the calculations of publication bias was of borderline significance (p = 0.05), what can suggest slight overestimation of the result. Prevalence of TC was quite high - about 4%. The risk of malignancy in acromegalic patients with TND was insignificantly higher than in control groups. There was also visible tendency that in newer studies thyroid disorders are reported more frequently – e.g. in studies published from 2008 TND occurred in about 65% of patients whereas in older studies – in about 54%; similar tendency can be observe in case of TCs – they were present in almost 6% of patients in papers published from 2008 and about 3% in older studies. This result is in line with suggestions, that the improving diagnostic and treatment of acromegaly extends the life duration what increases the prevalence of benign and malignant neoplasms. In the past, more patients died before neoplasms appeared or became clinically relevant [4]. The fact that our meta-analysis includes study performed in the period of over 50 years could be consider as limitation of this research. On the second, however there were numerable studies on the topic, amount of most reliable papers – prospective, including sex and age matched control groups and data both on the prevalnce of TC and TND is unsatisfactory. This fact is another limitation of this meta-analysis and it causes that confidence intervals of ORs and RRs are very wide, it also precludes detailed analysis of case – control studies in subgroups (e.g. newer vs. older studies).

It also calls attention that studies based on matching data from registers of acromegalic patients with data from cancer registers showed much lower frequency of TC than other, especially prospective studies, however in most cases insignificantly higher than expected [31], [32]. This discrepancy can be partially caused by inaccuracies in registers. On the other hand, these results may suggest, that TCs remained undiagnosed in great proportion.

In included studies the risk of malignancy for patients with TND was about 8%, what is in the range considered for general population [34]. Case – control studies also did not show significantly increased risk. However, the amount of studies is unsatisfactory; further researches are necessary to determine, if the risk of malignancy in acromegalic patients with TND is higher than in general population or if the frequency of TC is elevated proportionally to increased prevalence of TND.

However, many studies was published on the topic of increased risk of benign and malignant neoplasms in acromegaly, it remains controversial as results were often divergent. Among neoplasms, the increased prevalence of colon polyps and cancer seems to be most widely agreed, in large part thanks to meta-analysis performed by Rokkas et al. [5]. Comparing results of that meta-analysis with our outcomes, the risk of TC is elevated even more strongly than the risk of colon cancer (OR 7.9 *vs.* 4.4). Prevalence of these two malignancies seems to be similar in acromegalic patients, about 4.5%.

In conclusion, our meta-analysis proved that patients with acromegaly are at an increased risk of thyroid nodular disease and thyroid cancer. These results indicate, that periodic thyroid US examination and careful evaluation of eventual lesions should be important part of follow-up of acromegalic patients.

This study was performed with concorance with the PRISMA statement [35]. [S1].

## Supporting Information

Checklist S1 **PRISMA checklist.**
(DOC)
